# Retraction Note: Percutaneous pedicle screw fixation combined with selective transforaminal endoscopic decompression for the treatment of thoracolumbar burst fracture

**DOI:** 10.1186/s13018-020-02167-7

**Published:** 2020-12-30

**Authors:** Zhangheng Huang, Yuexin Tong, Zhiyi Fan, Chuan Hu, Chengliang Zhao

**Affiliations:** 1 Department of Spine Surgery, Affiliated Hospital of Chengde Medical University, Chengde, 067000 Hebei China; 2 Department of Orthopedics, The Affiliated Hospital of Qingdao University, Qingdao, 266000 Shandong China

**Retraction Note: J Orthop Surg Res (2020) 15:415**

**https://doi.org/10.1186/s13018-020-01946-6**

This article [1] has been retracted at the request of the authors. Following publication of the original article, the authors found errors in the counting and reconciling the data.

The Editor-in-Chief has offered the authors the opportunity resubmit their article with the correct results.

All authors agree to this retraction.

## References

[1] Huang Z (2020). Percutaneous pedicle screw fixation combined with selective transforaminal endoscopic decompression for the treatment of thoracolumbar burst fracture. J Orthop Surg Res.

